# REGULARITY OF RECURRENCE OF FIXED DRUG ERUPTION: A POINTER TO THE CAUSE

**DOI:** 10.4103/0019-5154.53176

**Published:** 2009

**Authors:** Betsy Ambooken, N Asokan

**Affiliations:** *From the Department of Dermatology and Venereology, Govt. Medical College, Trichur, Kerala, India. E-mail: joebetsy@sancharnet.in*

Sir,

A 35-year-old female presented with a recurrent skin lesion on the left thigh of four months duration. On examination, a well-defined erythematous plaque of size 5 × 3 cm with peripheral hyperpigmentation was observed. The lesion used to become erythematous and pruritic every month, a few days before her regular 28-day menstrual cycle; subsiding within 5–7 days leaving hyperpigmentation. A detailed history revealed that she used to take Tab. fluconazole 150 mg on the twenty-first day of her menstrual cycle for recurrent vaginal candidiasis as advised by her family doctor. A clinical diagnosis of fixed drug eruption (FDE) was made. She was treated with an antihistamine and topical corticosteroids with which the lesions became nonpruritic and hyperpigmented. Later, she gave a positive result to a re-challenge with fluconazole.

A 20-year-old male was seen with a recurrent pruritic skin lesion of 4 × 3 cm on the medial aspect of right ankle. The lesion used to become erythematous and pruritic once in almost exactly six months for the past 2 years. It used to subside within 3 days resulting in hyperpigmentation. A diagnosis of FDE was made. He used to take Tab. salbutamol 4 mg occasionally for bronchial asthma, but the onset of the lesions was not related to it. Further history taking revealed that he used to take Tab. levamisole 150 mg for 2 consecutive days at an interval of six months for boosting his ‘immunity’ as suggested by a local doctor and the onset of the skin lesions was related to these periods. After resolution of the lesion with antihistamine and topical corticosteroid, he was rechallenged with Tab. levamisole which gave a positive result.

Although there are reports of FDE to levamisole since 1991[1,2] and fluconazole since 1994,[3–6] recurrence of lesions at exactly regular intervals has not been reported. This striking feature led to the identification of the possible offender in both these patients. These cases are reported to highlight this interesting observation, which can give clue not only about the diagnosis, but also about the causative drug. In such instances, drugs such as fluconozole, which is commonly used as an intermittent treatment regimen may be suspected. The less commonly used drugs such as levamisole, which was prescribed in the second case for a vague indication, also could be the cause.
